# Discrimination of Breast Cancer Based on Ultrasound Images and Convolutional Neural Network

**DOI:** 10.1155/2022/7733583

**Published:** 2022-03-19

**Authors:** Rui Du, Yanwei Chen, Tao Li, Liang Shi, Zhengdong Fei, Yuefeng Li

**Affiliations:** ^1^Department of Ultrasound, Affiliated Hospital of Jiangsu University, Zhenjiang, China; ^2^Jiangsu Key Laboratory of Clinical Laboratory Medicine, School of Medicine, Jiangsu University, Zhenjiang, Jiangsu, China; ^3^Department of Nuclear Medicine, Nanjing First Hospital, Nanjing Medical University, Nanjing, Jiangsu, China; ^4^Department of Ultrasound, Affiliated Shuyang Hospital of Xuzhou Medical University, Shuyang, Jiangsu, China; ^5^Department of Medical Imaging, Affiliated Hospital of Jiangsu University, Zhenjiang, China

## Abstract

The aim of our study was to establish an artificial intelligence tool for the diagnosis of breast disease base on ultrasound (US) images. A deep learning algorithm Efficient-Det assisted US diagnosis method was developed to determine breast suspicious lesions as benign, malignant, or normal. Totally 1181 US images from 487 patients of our hospital and 694 publicly accessible images were employed for modeling, including 558 benign images, 370 malignant images, and 253 normal tissue images. The actual diagnosis results for the patients were determined by the biopsy or surgery. Efficient-Det was first retrained using an exclusive public breast cancer US dataset with transfer learning techniques. A blind test set consisting of 50 benign, 50 malignant, and 50 normal tissue images was randomly picked from the patients' images as the independent test set to test its searching ability on suspicious tumor regions. Furthermore, the confusion matrix and classification accuracy were employed as evaluation metrics to select the optimal classification models. Efficient-Det has demonstrated remarkable progress in general image recognition tasks with specific advantages of locating and identifying tumor areas simultaneously. Compared to the manual method (mean accuracy: 95.3% and 60 s per image) and traditional feature engineering method (mean accuracy: 90% and 15 s per image), our Efficient-Det has the capability of providing a competitive (mean accuracy: 92.6%) and fast (0.06 s per image) classification result. The deployment of Efficient-Det in our local breast cancer discrimination task exhibits specific applicability within real clinical workflows.

## 1. Introduction

Breast cancer risk is the second leading cause of death among women in the world, but precise detection can provide an opportunity for timely treatments [1]. Among the various detection methods, B-mode ultrasound screening technology is favored and recommended as a routine diagnostic tool due to its low cost and fast imaging [2]. Although breast ultrasound imaging can characterize the suspicious tumor areas of the breast tissue, massive daily image analysis aggravates the burden of clinical radiologists [3]. Furthermore, the inconsistency of different radiologists on the same image may lead to serious false-positive problems, thereby delaying the effective treatments [4].

Currently, different computer-aided diagnosis (CAD) systems have been developed to standardize and accelerate the diagnostic procedure [5]. Traditional CAD systems usually consist of two steps: segmentation and identification of suspicious breast tumor regions. In the segmentation section, interactive, semiautomatic, and even fully automatic methods are implemented to generate cancer contour segmentations [6]. Next, quantitative metrics, including morphological or texture features of cancer regions, are extracted following professional instructions [7]. After feature engineering, various classification algorithms, such as linear discriminant analysis (LDA) and support vector machines (SVMs), are then employed for the discrimination of cancer types [8]. However, the performance of the two-stage cancer detection approach largely depends on the coincidence degree of feature engineering and classification algorithms. The prediction of breast cancers in a one-stage fashion might be the trend, which has the advantage of providing flexible and faster diagnostic results.

In recent years, deep learning (DL) algorithms, particularly convolutional neural networks (CNNs), have gained extensive attention for classification tasks in different scenes owing to their outstanding performance [9]. Unlike complicated handcrafted feature engineering, the convolutional neural network (CNN) architecture is capable of automatically extracting repeatable and accurate representative deep features [10]. As a consequence, major medical and governmental organizations are seeking efficient artificial intelligence (AI) algorithms to achieve automated clinical applications [11]. Various CNN algorithms, such as ResNet, U-Net, and DenseNet, have been redesigned to directly inspect cancer images [10], to locate precise cancer areas [12] or to mimic the human decision-making process in diagnosis [13], and have shown excellent performance. Although AI approaches show promise in breast cancer image analysis, the expensive soft and hardware costs limit their clinical applicability [14]. Recently, to reduce dependence on models with abundant complex parameters, more research has focused on designing a tiny and ingenious AI algorithm for rapid detection. Efficient-Det is gaining increasing attention due to its remarkable performance based on the tiny scale of parameters [15]. The high standard dataset is becoming another technical obstacle, especially for developing nations. Large-scale investigation programs are hard to implement, resulting in a shortage of high-quality labeled datasets [16]. To fulfill this technical gap, transfer learning techniques might be a welcomed choice to retrain the general CNN architecture for the image classification task of local breast cancer cases [17].

In this paper, we modified Efficient-Det, which is a lite architecture proposed by Tan et al. [15], and used it to differentiate benign breast masses, malignant breast masses, and normal breast tissue based on two-dimensional gray scale ultrasound images. In addition, expert radiologist methods and feature engineering-based classification methods were also conducted for comparison. We then explored the interpretability of AI methods in improving the accuracy of discriminating breast cancer images. The clinical applicability of Efficient-Det in breast tumor classification is also discussed.

## 2. Material and Methods

### 2.1. Study Design and Participants

**Figure 1:**
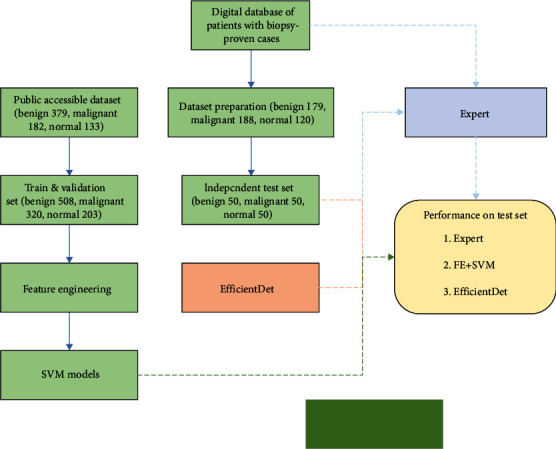
Flow chart of the development procedures for the deep learning model for breast tumor discrimination.

We carried out a retrospective, single center, and diagnostic study by searching the images of 986 female patients who underwent breast ultrasound examination in the ultrasound image database in our hospital from June 2015 to June 2020. According to the exclusion criteria, 499 patients were excluded according to the following criteria: (1) any preoperative intervention and treatment (such as radiotherapy and chemotherapy) before ultrasound examination and (2) the mass showed unclear or no visible region of interest (ROI) on the sonogram. Clinical data included age, family history of breast cancer, menopausal status, and pathological type. Image quality was assessed by two radiologists with 5 and 10 years of breast imaging experience. The flow chart describing the research process is shown in Figure 1. To enrich the dataset, a publicly accessible dataset including 379 benign, 182 malignant, and 133 normal tissue images was employed for model training (https://www.kaggle.com/anaselmasry/datasetbusiwithgt/version/1). Totally 1181 images were used for data analysis in the present work, containing 558 benign images, 370 malignant images, and 253 normal tissue images.

### 2.2. Image Acquisition and Preprocessing

**Figure 2:**
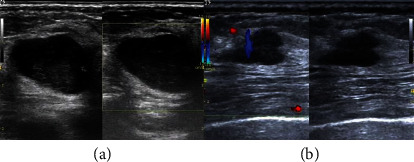
Breast tumor images acquired with two-dimensional gray scale ultrasound and color Doppler ultrasound: (a) malignant tumor image; (b) benign tumor image.

The two-dimensional gray scale ultrasound images of breast masses were obtained from 10 different ultrasound machines from four manufacturers, and detailed information is presented in Table S1. All patients were examined with a 7–12 MHz linear probe. The transducer was directly applied to the skin surface to check the inner and outer quadrants of the breast. Then, a scan was performed on the radial and antiradial planes associated with the nipple or the sagittal and transverse planes, starting from the inner upper quadrant of the breast and then slowly moving to the outer quadrant to obtain the sagittal image. Then, the transducer was moved under the breast, and the scan was repeated until the entire breast was examined. The benign and malignant images are shown in Figure 2.

### 2.3. Image Preprocessing

For Efficient-Det, the open-source software LabelImg was used to label the tumor ROI with annotations (https://github.com/tzutalin/labelImg). Then, the preprocessed images coupled with annotations were saved and yielded for the loop training of Efficient-Det.

For expert radiologists, original test images were directly presented coupled with digital patient history.

For feature engineering- (FE-) based methods, the cancer regions of interest (ROIs) were manually cropped by an expert-defined method, which covered the entire tumor area and boundary close to the tumor margin. Next, the tumor ROI segmentations were fed to the automated program for feature extraction. The detailed morphological features including area, eccentricity, and orientation. Second, based on these features are summarized in Table S2, as well as the corresponding mathematical definitions.

### 2.4. Efficient-Det Algorithm

**Figure 3:**
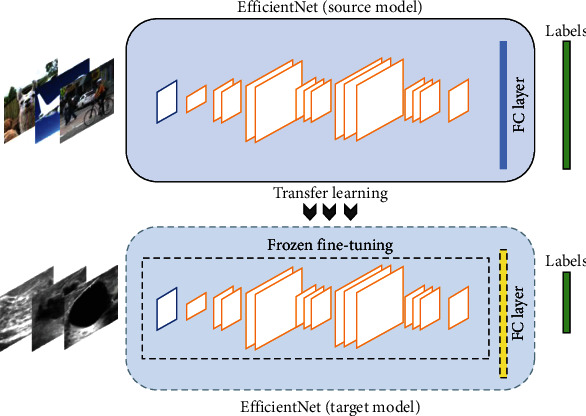
The procedure of fine-tuning Efficient-Net (Efficient-Det core algorithm) using the transfer learning technique. Different from the structure of Tan's workflow (be reserved for feature extraction), the original prediction layer in Efficient-Net was changed into the categories of benign, malignant, and normal scores (the rightest label bar), and the hyperparameters of the modified Efficient-Det were next updated to satisfy for breast cancer classification using transfer learning strategy.

The Efficient-Det algorithm presented here was a one-stage architecture that is capable of providing precise segmentation and accurate classification simultaneously. The efficiency of this new model was significantly improved by conducting systematic parameter optimization including weighted bidirectional feature pyramid networks (BiFPNs) and the compound scaling strategy of depth, width, and resolution of the network. Efficient-Det-B0 was selected here, which has the tiniest model size but can achieve competitive accuracy. The network parameters and the structure of Efficient-Det-B0 are presented in detail in Table S3 and Figure 3, respectively. The mobile inverted bottleneck (MBCov) designed by MobileNet-V2 was used for baseline construction, which could extract deeper features with less computation. After feature extraction, the final prediction layer was the categories of benign, malignant, and normal scores. A transfer learning strategy was employed to modify Efficient-Det for breast tumor classification. First, the parameters of Efficient-Det were initialized with values derived from COCO dataset pretraining. Then, data augmentation, including shearing, rescaling, and flipping, was applied to each breast tumor image during model training. Specific training skills, such as early stopping, learning rate decay, and freeze fine-tuning, were conducted to speed up model training. The Efficient-Det loss is defined in Equation (1), which is the summation of smooth_*L*1_ and Focal Loss. smooth_*L*1_ is the box regression regulation function commonly used on object detection systems, which is defined in Equation (2), and *x* denotes the error between the predicted value and true value. Loss_FL_ was proposed by He et al. to solve the positive-negative imbalance problem that occurs in object detection [18]. It is defined in Equation (3), where *y*′ denotes the output after the activation function, *α* denotes the balancing factor, and *γ* (gamma) is used to adjust the weight decrease rate of simple samples.

(1) $$\begin{array}{c} \text{Loss}={\text{smooth}}_{L1}+{\text{Loss}}_{\text{FL}}, \end{array}$$

(2) $$\begin{array}{c} {\text{smooth}}_{L1}\left(x\right)=\left\{\begin{array}{cc} 0.5x^{2} & \left|x\right|<1\\ \left|x\right|-0.5 & \text{otherwise} \end{array},\right. \end{array}$$

(3) $$\begin{array}{c} {\text{Loss}}_{\text{FL}}=\left\{\begin{array}{cc} -\alpha {\left(1-y^{\prime }\right)}^{\gamma }\;\log y^{\prime } & y=1\\ -\left(1-\alpha \right)y^{\prime \gamma }\log\left(1-y^{\prime }\right) & y=0 \end{array}\right.. \end{array}$$

### 2.5. Model Training

In the Efficient-Det training, 150 samples (namely, 50 benign images +50 malignant images +50 normal tissue images) were randomly picked from the patients' images from our hospital. They formed a blind test set used for to examine the Efficient-Det model searching ability on suspicious tumor regions and the predicative ability. The remaining 1031 images were shuffled for model training and validation with a split ratio value of 0.75, and the weighted split strategy for different classes was conducted to avoid imbalance issues.

For the expert diagnosis, only the selected 150 samples in Efficient-Det model blind test set were used to examine the accuracy of expert diagnosis. The discriminant ability for the expert diagnosis was determined by results from biopsy or surgery. For the feature engineering, the SVM classifier with a linear kernel (default parameter) was then employed to achieve automated discrimination of benign and malignant tumors based on morphological features. Because there were no morphological features for the normal tissue images, only benign images and malignant images were used for modeling. The 50 benign images and 50 malignant images in the Efficient-Det model blind test set were also used for bind test in the FE-SVM model.

### 2.6. Evaluation Metrics

In this study, a confusion matrix and a classification accuracy were employed as evaluation metrics [19]. The confusion matrix lists all actual samples in columns and predicted samples in rows. The classification accuracy is defined in Equation (4), where TP, TN, FP, and FN denote true positive, true negative, false-positive, and false-negative samples, respectively. The mean accuracy defined in Equation (5) was employed to quantify the performance of each classification model.

(4) $$\begin{array}{c} \text{Accuary}=\frac{\text{TP}+\text{TN}}{\text{TP}+\text{FP}+\text{FN}+\text{TN}}, \end{array}$$

(5) $$\begin{array}{c} \text{Mean Accuracy}=\frac{1}{k}\overset{k}{\underset{i=0}{\sum }}{\text{Accuracy}}_{k}. \end{array}$$

## 3. Results

### 3.1. Performance Comparison of Different Classification Methods

**Figure 4:**
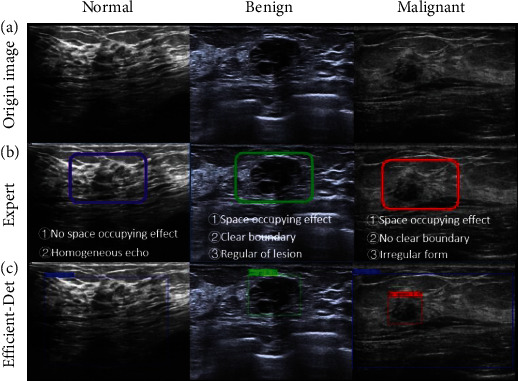
Classification results on different images between experts and Efficient-Det: (a) origin images; (b) identification process and results of experts; (c) identification results of Efficient-Det.

Table 1 summarizes the results of all classification methods. The expert diagnosis achieved the highest classification accuracy (mean accuracy 95.3%), followed by Efficient-Det (mean accuracy 92.6%) and SVM classifier with feature engineering techniques (mean accuracy 90%). In addition, the Efficient-Det model had the shortest time-consuming (0.06 s per image). The classification results in Figure 4 show that our Efficient-Det may intuitionistically present breast cancer diagnosis results compared to other methods. An excellent localization performance suggests that our Efficient-Det can precisely select suspicious tumor regions for further assessment. Notably, additional cancer identification probabilities are given to support the inferred results. Beyond detailed prediction probabilities, the Efficient-Det could obviate the need for double reading and thereby may have many practical benefits.

### 3.2. Confusion Metrics of Prediction Results of Different Classifiers

**Figure 5:**
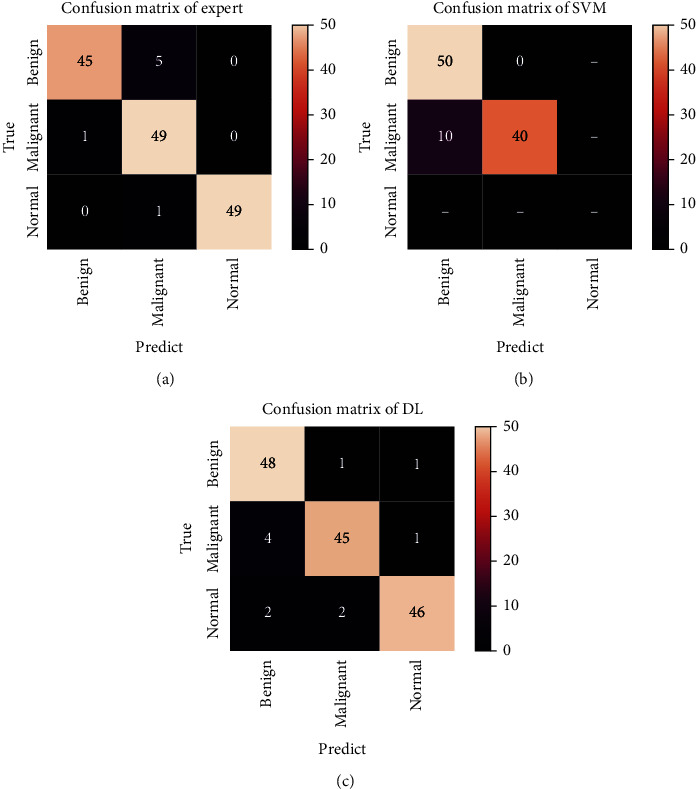
Confusion metrics of different classifiers: (a) expert; (b) SVM classifier; (c) DL classifier.

The prediction results of three classification methods for benign lesions, malignant lesions, and normal tissues were presented in the confusion matrix (Figure 5). Five images of benign lesions were misclassified as malignant as shown in Figure 5(a) by experts, while their predictions of malignant lesions and normal tissues were consistent with the real results. SVM classifier performed well in the prediction of benign lesions, but there were 10 cases of misjudgment in malignant lesions as shown in Figure 5(b). The accuracy of the Efficient-Det model was higher than that of experts (48/50 vs. 45/50) in identifying benign lesions but lower than that of experts in identifying malignant lesions and normal tissues (45/50 vs. 49/50; 46/50 vs. 49/50). Compared with SVM classifier, the Efficient-Det model had slightly lower accuracy in identifying benign lesions (48/50 vs. 50/50), while it was more accurate in identifying malignant lesions (45/50 vs. 40/50) (Figure 5(c)).

### 3.3. False-Positive Classification Cases of All Classification Models

**Figure 6:**
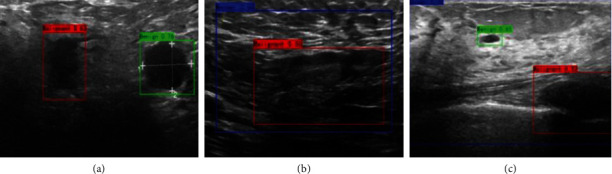
False-positive classification cases: (a) real malignant cases with benign referral; (b) a small dark region was identified as a benign tumor; (c) real tissue image with benign and malignant predictions.

The minimum false-positive cases of all classification models centered around ambiguous images, which are presented in Figure 6. The malignant lesion was misclassified as a benign image with a 0.76 confidence score (Figure 6(a)). The gland ROI (red rectangle) was misclassified as a malignant image with a confidence score of 0.56 by Efficient-Det. In contrast, another gland ROI (blue rectangle) was captured, which produced a different explanation (normal image confidence score: 0.50) (Figure 6(b)). Efficient-Det provided three different explanations in an image with only one benign lesion ROI, while only one was correct (green rectangle) with a confidence score of 0.61. The ROI of cross section of the rib (red rectangle) was misclassified as a malignant image, while the small benign lesion was not effectively recognized, resulting in the ROI (blue rectangle) of the normal area being captured (Figure 6(c)).

### 3.4. Grad-Cam Visualization of Convolutional Features

**Figure 7:**
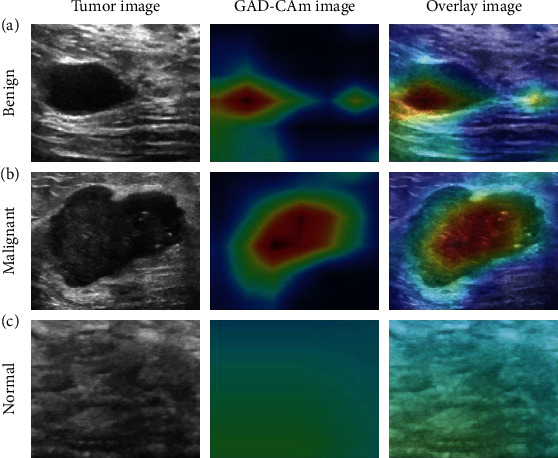
Grad-Cam visualization of convolutional features: (a) Grad-Cam performed on predicting benign tumor image; (b) Grad-Cam performed on predicting malignant tumor images; (c) Grad-Cam performed on predicting normal tissue image.

We intuitively presented the recognition pattern of the DL model by generating heat maps, including Grad-Cam feature heat map, and the overlay images (Figure 7): The Efficient-Det activated the greatest predictive regions of the tumor with red and yellow, while the weaker predictive regions were green and blue. As shown in Figures 7(a) and 7(b), when visualized for tumors, the suspicious regions were highlighted. Moreover, the Grad-Cam features showed that Efficient-Det was interested in the center region inside the tumor (red colors highlight from overlay images), which may be important for predicting the particular variety of tumors. In Figure 7(c), Grad-Can features show that it failed in detecting any suspicious tumor ROIs, which supports the Efficient-Det to provide a “normal” decision.

## 4. Discussion

In this study, we present an intelligent classification method (Efficient-Det) that achieved competitive results with expert radiologists on a clinical task of ultrasound breast cancer classification. Although manual and traditional ML classification methods show encouraging results, these studies have relied on plenty of enriched experience or professional, handcrafted engineering features. For instance, when human readers evaluate breast cancer, they may obtain access to digital patient history to confirm the final referral decision. From Table 1, it seems acceptable that only 60 s was needed when manually diagnosing each image, but a flood of ultrasound breast cancer images was generated daily, and labor shortages became an unavoidable challenge. In contrast, the ML method offers a huge improvement because it provides automatic assessment. However, the establishment of feature engineering will cost considerable manpower and time. Consequently, only a few advanced medical institutions have the ability to afford an expensive classification system based on complicated feature engineering. In addition, the traditional ML method is not interface-friendly for clinical radiologists; inferred results were directly present without cancer location.

Although an expert can classify different types of breast cancers with the highest accuracy, the time cost of 60 seconds per image is extraordinary for a clinical radiologist. Long-term high-intensity image inspection will inevitably lead to persistent anxiety, stress, and low accuracy. Compared to the expert method, the two-stage traditional CAD method, which employs an SVM classifier with feature engineering, can eliminate repetitive and tedious human inspection. Although feature engineering and SVM classifiers can currently be implemented separately using program automation, additional operations such as feature sorting and standardization are needed. Furthermore, solely based on morphological features, only benign and malignant tumors can be binary classified, and normal tissue images cannot be effectively recognized. Recent searches believe that texture feature extraction can enrich feature engineering functions, thereby improving the classification accuracy; however, the time cost of processing per image will be significantly increased. Our one-stage Efficient-Det achieves a balanced result, with a high classification accuracy of 92.6%, similar to expert behavior, and short time (0.06 s per image). Despite the huge computational resources required for optimizing the Efficient-Det model, once training on the model is complete, it could be deployed in the cloud for automated classification of breast tumors.

However, these comparisons are not without limitations. Especially for traditional ML classifiers, the normal tissue images were not fed to feature engineering. In the past, the specific gray level-gradient cooccurrence matrix (GLCM) technique was conducted to quantify normal tissue images [20]. Since many studies have provided evidence that CNN features are superior to handcrafted GLCM features, this time-consuming and tedious FE was not conducted here. Another limitation comes from the interpretation of different classification models. Usually, human readers add the symptom description to support their screening decisions, which could be traced from the patient's digital history. Although the ML and DL methods exhibit a tradeoff between accuracy and simplicity, both only provide the predicted probability scores as inference results. In ML methods, the first feature engineering component provides a naturally initial explanation when designing the handcrafted rule to quantify tumor regions, where classical rule-based methods are highly enriched in interpretation but may not be robust enough for characterization. The power of Efficient-Det comes from the deep CNN architecture by stacking greater abstraction (more deep convolutional layers) and tighter integration (back-propagation derivation and end-to-end training) using advanced establishment techniques. Consequently, while Efficient-Det enables superior performance to other methods, the lack of decomposability into inside individual components makes it a regrettable “black box,” which is hard to interpret.

To increase the interpretation of large-scale CNN models such as Efficient-Det, the gradient-weighted class activation mapping (Grad-Cam) technique was employed here to visualize the “black box” [21]. Grad-Cam can produce “visual explanations” for model decisions by generating a coarse localization map highlighting the important regions when predicting the target tumors. As shown in Figure 7, we present the original tumor image, corresponding Grad-Cam feature heat map, and the overlay images. The Grad-Cam feature heat map was calculated by extracting the gradient information from the last convolutional layer, in which a particular decision value of interest is reserved. Furthermore, to present the high-resolution and class-discriminative Grad-CAM visualizations, pixel-space gradients and locations are fused. Specifically, ReLU activation is used to the linear combination of weighted feature maps to force the Grad-Cam feature to focus on the features which have a positive influence on the class of interest. By adopting this strategy, the positive pixel intensity of targeted regions on Grad-Cam is increased, while negative pixels belonging to other categories are weakened. As a result, important regions of the image that correspond to any decision of interest are visualized in high-resolution detail when the image contains evidence for suspicious regions. As shown in Figures 7(a) and 7(b), when visualized for tumors, the suspicious regions were highlighted. Moreover, the Grad-Cam features showed that Efficient-Det was interested in the center region inside the tumor (red colors highlight from overlay images), which may be important for predicting the particular variety of cancer tumors. In Figure 7(c), Grad-Can features show that it failed in detecting any suspicious tumor ROIs, which supports the Efficient-Det to provide a “normal” decision.

## 5. Conclusion and Future Perspectives

We present the evidence of Efficient-Det's capability to detect and identify breast tumors based on two-dimensional gray scale ultrasound images in this study. We retrained Efficient-Det using an exclusively public ultrasound dataset and then measured performance on our local breast cancer dataset. In this context, Efficient-Det (mean accuracy 92.6%) outperforms traditional FE-based CAD methods (mean accuracy 90%) by a smaller margin. The time cost per image for Efficient-Det was excellent, only at 0.06 s. Although expert radiologists achieved a higher mean accuracy score (95.3%) than Efficient-Det, a huge time cost was required for deciding each image (60 s). Our research suggests that in future clinical deployments, Efficient-Det might offer competitive diagnostic results in a shorter time. Furthermore, Efficient-Det may benefit from fine-tuning with increasing local data. Since only limited data are currently provided, extra data could help Efficient-Det build a strong baseline for anti-false-positive cases, thereby providing robust and precise diagnostic results. Although early stage detection of breast cancer is more clinical, it is also challenging. Hence, in order to construct the preliminary structural framework of the diagnostic model, more distinctive parameters from benign and malignant lesions were preferentially included and analyzed, which provided a parameter basis for the subsequent construction of early stage detection model.

AI systems tend to be invasive rather than the current routine procedure. Although the optimal use of the AI method within clinical workflows remains to be determined, current research suggests that AI technology exhibits a specific advantage in simplifying the clinical procedure (such as unnecessary biopsies) and improving diagnostic accuracy. Since US technology is fast and widely adopted in screening breast cancer, AI-assisted US detection systems are preferred by more researchers. Additionally, it is necessary to explore intelligent and tiny AI algorithms for cloud deployment. We believe that our lite Efficient-Det research will provide a profound understanding of applying intelligent algorithms for breast tumor classification and valuable insights to researchers in this field.

## Figures and Tables

**Table 1:** Results from different classification methods.

Classification methods	Classification accuracy			Mean accuracy	Time cost per image
	Benign	Malignant	Normal		
Expert	90% (45/50)	98% (49/50)	98% (49/50)	95.3% (143/150)	60 s
FE + SVM	100% (50/50)	80% (40/50)	—	90% (90/100)	15 s
Efficient-Det	96% (48/50)	90% (45/50)	92% (46/50)	92.6% (139/150)	0.06 s

Expert: expert radiologist manual decisions; FE + SVM: feature engineering with support vector machines; Efficient-Det.

## Data Availability

Dataset is available under reasonable request.

## Supplementary Materials

Supplementary Materials Table S1: detailed B-mode ultrasound breast cancer image acquisition systems. Table S2: list of extracted morphological features. Table S3: list of parameters of Efficient-Det-B0.
