# Supplementary material for: Discrimination of Breast Cancer Based on Ultrasound Images and Convolutional Neural Network
Source: J Oncol. 2022 Mar 19;2022:7733583. doi: 10.1155/2022/7733583 (PMC8957444; doi:10.1155/2022/7733583)
Supplement: Supplementary Materials — Table S1: detailed B-mode ultrasound breast cancer image acquisition systems. Table S2: list of extracted morphological features. Table S3: list of parameters of Efficient-Det-B0. [file 7733583.f1.docx]

**Supplementary Materials**

1. **B-mode ultrasound acquisition systems**

The ultrasound breast cancer image acquisition systems for this research were present in Table S1. Mention of these commercial products is solely for the purpose of providing specific information and does not imply recommendation or endorsement.

**Table S1**. Detailed B-mode ultrasound breast cancer image acquisition systems

| Manufacture | Country | Name |
| --- | --- | --- |
| GE Healthcare | United States | LOGIQ E9 |
|  |  | LOGIQ E8 |
|  |  | VOLOUSION E10 |
|  |  | VOLOUSION 730 |
| Esaote | Italy | MYLAB TWICE |
|  |  | MYLAB CLASS C |
|  |  | MYLAB 90 |
| Toshiba | Japan | APLIO 500 |
|  |  | APLIO 400 |
| Siemens | German | SIEMENS 300 |

1. **Morphological features of breast cancer tumor**

High frequency ultrasound has excellent resolution for superficial organs such as mammary gland. The common ultrasonic morphological features of breast cancer tumor were summarized in Table S2.

**Table S2**. List of extracted morphological features.

| Morphological  Feature Name | Morphological Properties | Description |
| --- | --- | --- |
| Area | The area of tumor | Number of pixels of the tumor region |
| Eccentricity | The eccentricity value of the tumor | The eccentricity is the radio of the focal distance over the major axis length |
| Max length | Major axis length | The major axis length is the max distance passing through the foci of the tumor |
| Min length | Minor axis length | The minor axis length is the minor distance passing through the foci of the tumor |
| Min Max ratio | Min length / Max length | The ratio value of minor axis length over the major axis length |
| Diameter | Equivalent diameter | The diameter of a circle with the same area as the region |
| Orientation | Orientation value | The angle value between the horizontal axis and the major axis of the tumor |
| Extent | Extent value | Ratio of pixels in the region to pixels in the total bounding box |
| Perimeter | Perimeter value | Perimeter is defined as the boundary that surround the tumor |

1. **Morphological features of breast cancer tumor**

The ML algorithm was coded by Python (version 3.7, Google Inc., San Francisco, CA, USA), the feature engineering and classifier support package could be reached in website https://scikit-image.org/ and https://scikit-learn.org/stable/, respectively.

**Table S3**. List of parameters of Efiicient-Det-B0.

| Stage | Operator | Resolution Size | Layer Numbers |
| --- | --- | --- | --- |
| 1 | Conv 3×3 | 224×224 | 1 |
| 2 | MBConv1, 3×3 | 112×112 | 1 |
| 3 | MBConv6, 3×3 | 112×112 | 2 |
| 4 | MBConv6, 5×5 | 56×56 | 2 |
| 5 | MBConv6, 3×3 | 28×28 | 3 |
| 6 | MBConv6, 5×5 | 14×14 | 3 |
| 7 | MBConv6, 5×5 | 14×14 | 4 |
| 8 | MBConv6, 3×3 | 7×7 | 1 |
